# Loneliness trajectories and psychological distress in youth: Longitudinal evidence from a population‐based sample

**DOI:** 10.1111/bjdp.12533

**Published:** 2024-11-16

**Authors:** Emma M. Kirwan, Martina Luchetti, Annette Burns, Páraic S. O'Súilleabháin, Ann‐Marie Creaven

**Affiliations:** ^1^ Department of Psychology University of Limerick Limerick Ireland; ^2^ Health Research Institute University of Limerick Limerick Ireland; ^3^ Department of Behavioral Sciences and Social Medicine Florida State University Tallahassee USA; ^4^ Health Research Board Dublin Ireland

**Keywords:** adolescence, loneliness, psychological distress, trajectory, understanding society

## Abstract

This pre‐registered secondary analysis aimed to examine distinct longitudinal loneliness trajectories in youth and whether these trajectories were associated with psychological distress at final follow‐up in the UK Household Longitudinal Study. Participants (*N* = 827, 55.1% female, Time 1: *M* ± *SD* = 16.50 ± 0.50 years) provided data during Waves 9, 10 and 11. K‐means longitudinal clustering analysis was used to identify clusters of participants with distinct loneliness trajectories across measurement waves. We identified four clusters demonstrating distinct trajectories of loneliness: stable low (40.7%), stable high (20.6%), moderate decreasing (19.6%) and low increasing (19.1%). Compared to ‘stable low loneliness’, ‘stable high’ and ‘low increasing’ loneliness clusters were significantly associated with psychological distress at Wave 11 following adjustment for sex, ethnicity, parent's highest educational achievement and Wave 9 psychological distress. The current study offers an important contribution to the literature on patterns of youth loneliness and mental health consequences.

**Statement of contribution**

**What is already known about this subject?**

- Loneliness is particularly prevalent in youth, which is also a critical period for mental health.
- Existing research has identified longitudinal loneliness trajectories in youth.
- Fewer works have considered outcomes associated with loneliness trajectories in youth.

**What the present study adds?**

- This study identified distinct longitudinal trajectories of loneliness and demonstrated associations with psychological distress in youth.
- Stable, relatively high loneliness levels during youth may be detrimental to mental health.

## INTRODUCTION

Loneliness is the unpleasant emotional state experienced when a person perceives a discrepancy between their actual and desired social relationships, either in quantity or quality (Peplau & Perlman, 1982). Loneliness is an important issue across the lifespan, not only because it involves potentially distressing feelings but also because it is a well‐established risk factor for poorer physical and mental health outcomes, including depression, cardiovascular disease and all‐cause mortality (Hawkley & Cacioppo, 2010; Holt‐Lunstad et al., 2015; Leigh‐Hunt et al., 2017; Luo et al., 2012; Park et al., 2020; Wang et al., 2023). Despite different and overlapping definitions of adolescence (e.g., 10–19 years; World Health Organization, 2001) and emerging adulthood (e.g., 18–25 years; Arnett, 2000), research has consistently reported high levels of loneliness among youth (i.e., 15–25 years) (Pyle & Evans, 2018; Qualter et al., 2015; Victor & Yang, 2012). Late adolescence and emerging adulthood typically involve developmental changes, social transitions, identity exploration and increased autonomy and individuation, which can lead to shifts in social relationships and a greater risk of loneliness (Arnett, 2024; Laursen & Hartl, 2013). Notably, youth is also a critical period for the emergence of mental health issues, including mood disorders (Solmi et al., 2022).

The impact of loneliness on mental health does not stem from an objective lack of social contacts, but rather from the distressing experience of perceiving one's social relationships as inadequate (Hawkley & Cacioppo, 2010). According to the evolutionary theory of loneliness, transient loneliness serves an adaptive function where short‐term emotional distress motivates reconnection. Failure to reconnect can precipitate a negative feedback loop where heightened risk perception and social avoidance exacerbate loneliness and associated psychological distress (Cacioppo et al., 2006). Chronic loneliness may be a precursor to more significant mental health issues (Hawkley & Cacioppo, 2010), which often emerge during youth and persist into adulthood (Kessler et al., 2007). Studies mostly assess ‘chronicity’ by examining the frequency of loneliness using self‐reported measures administered at one point in time. Yet, some studies have assessed loneliness over time, indicating that even transient loneliness is associated with poor health outcomes (Martín‐María et al., 2020, 2021). These studies have tended to focus on middle‐aged and older adulthood, although loneliness also peaks in youth (Luhmann & Hawkley, 2016; Victor & Yang, 2012). Emerging adults' own perspectives also suggest that the distinction between transient and chronic loneliness may be particularly relevant, given that transient experiences are common and may be tied to normative development during this life stage (Kirwan et al., 2023). Therefore, understanding associations between both transient and chronic loneliness and indicators of poor mental health during youth is imperative.

Notably, not all youth follow the same longitudinal pattern of development and change in loneliness. A growing research base has suggested interindividual differences in patterns of loneliness in adolescence and emerging adulthood reporting between 2 and 5 clusters of distinct trajectories (Hutten et al., 2021; Ladd & Ettekal, 2013; Lin & Chiao, 2022; Qualter et al., 2013; Schinka et al., 2013; Vanhalst, Goossens, et al., 2013; Vanhalst, Luyckx, et al., 2013). These studies consistently demonstrate that the largest amount of youth (37%–63%) experience stable low levels of loneliness over time. Some have reported a smaller group experiencing persistent, high levels of loneliness, indicating a chronic pattern characterised by poorer mental health (Ladd & Ettekal, 2013; Qualter et al., 2013; Vanhalst, Goossens, et al., 2013; Vanhalst, Luyckx, et al., 2013). Patterns of change, characterised by increasing or decreasing loneliness levels over time, have also been reported (Hutten et al., 2021; Ladd & Ettekal, 2013; Lin & Chiao, 2022; Qualter et al., 2013; Vanhalst, Goossens, et al., 2013; Vanhalst, Luyckx, et al., 2013) and may be associated with poorer mental health outcomes. Across the ages of 15–20 years, Dutch youth with a low‐increasing loneliness trajectory reported higher levels of depressive and anxiety symptoms compared to a low stable trajectory (Vanhalst, Goossens, et al., 2013; Vanhalst, Luyckx, et al., 2013). Similarly, Hutten et al. (2021) found that compared to stable low loneliness, Dutch adolescents with high‐decreasing and low‐increasing loneliness trajectories had a higher risk of depression at age 21. While this small number of studies have examined patterns of loneliness across late adolescence and emerging adulthood suggesting links to poorer mental health, there is still much to be done to examine the consequences of loneliness during this critical life stage and previous trajectory research needs replication in other samples. Loneliness and psychological distress have been linked in previous research with late adolescents (Shevlin et al., 2013), where loneliness was measured at a single assessment. The current study extends the existing literature by examining longitudinal trajectories of loneliness and their associations with psychological distress in youth in the United Kingdom.

### Aims

This pre‐registered secondary data analysis, using three annual assessments of loneliness in a sample of youth from the UK Household Longitudinal Study (UKHLS), aimed to explore patterns of loneliness across late adolescence and examine whether trajectories of loneliness were associated with psychological distress. We expected that youth demonstrating a trajectory characterised by consistently high levels of loneliness would report greater psychological distress. Given that transient loneliness may be associated with poorer outcomes (Martín‐María et al., 2020, 2021), we also explored whether youth demonstrating a pattern characterised by transient loneliness, compared to consistently low levels of loneliness, were at risk of psychological distress. Taken together, our study aimed to provide a comprehensive exploration of loneliness trajectories and psychological distress; in the interest of completeness, we also examined if loneliness trajectories were associated with psychological distress cut‐off scores.

## MATERIALS AND METHODS

### Participants and procedure

The UKHLS (also known as Understanding Society Survey; University of Essex, 2022) is an ongoing, nationally representative survey, established in 2009 with approximately 40,000 households (for overview see, Buck & McFall, 2012). Ethical approval for UKHLS was granted by the University of Essex Ethics Committee (Understanding Society, n.d.).

We used UKHLS data from Waves 9 (collected from January 2017 to May 2019), 10 (collected from January 2018 to May 2020) and 11 (collected from January 2019 to May 2021), as these were the only waves for which loneliness data were available. The sample consisted of participants aged 16 and 17 years at Wave 9, who had valid data on the loneliness measure. To be included in our analytical sample, participants need to have completed one follow‐up assessment (either at Wave 10 or 11). We excluded ‘proxy’ respondents (*n* = 57) as these were missing data for the main study variables, including loneliness and psychological distress. In the UKHLS, a ‘proxy’ response is where a participant was unavailable, and another household member responded to a short factual questionnaire on their behalf. We also excluded one participant whose response was recorded as inconsistent for the main confounding variable of sex. The analyses were based on *N* = 827 (Wave 9 *M* ± *SD* = 16.50 ± .50 years; Wave 10 *M* ± *SD* = 17.48 ± .54 years; Wave 11 *M* ± *SD* = 18.46 ± .57 years). Compared to those with available data, youth not included in final analyses (*n* = 172) were more likely to be Black or other ethnicity (Cramer's *V* = .100, *p* = .019). No significant differences were observed for sex (*p* = .461), Wave 11 psychological distress (*p* = .482) or Wave 9 loneliness (*p* = .315). See Table 1 for descriptive statistics.

**TABLE 1:** Demographic characteristics for total sample and mean scores for key variables split across loneliness clusters.

Variable	Total sample (*N* = 827)	Stable low (*N* = 337)	Stable high (*N* = 170)	Moderate decreasing (*N* = 162)	Low increasing (*N* = 158)
Gender, *N* (%)					
Male	371 (44.9%)	171 (46.1%)	76 (20.5%)	58 (15.6%)	66 (17.8%)
Female	456 (55.1%)	166 (36.4%)	94 (20.6%)	104 (22.8%)	92 (20.2%)
Ethnicity, *N* (%)					
White UK	551 (66.6%)	199 (36.1%)	128 (23.2%)	110 (20.0%)	114 (20.7%)
White other	42 (5.1%)	17 (40.5%)	13 (31.0%)	6 (14.3%)	6 (14.3%)
Black	37 (4.5%)	14 (37.8%)	8 (21.6%)	7 (18.9%)	8 (21.6%)
Other	197 (23.8%)	107 (54.3%)	21 (10.7%)	39 (19.8%)	30 (15.2%)
Parent's highest educational qualification					
No qualification	30 (3.8%)	16 (5.0%)	2 (1.3%)	6 (4.0%)	6 (3.9%)
Other qualification	47 (6.0%)	21 (6.6%)	9 (5.7%)	4 (2.6%)	13 (8.4%)
GCSE or equivalent	145 (18.6%)	66 (20.8%)	31 (19.6%)	24 (15.9%)	24 (15.6%)
A‐level or equivalent	149 (19.1%)	58 (18.2%)	32 (20.3%)	30 (19.9%)	29 (18.8%)
Higher degree	410 (52.5%)	157 (49.4%)	84 (53.2%)	87 (57.6%)	82 (53.2%)
W11 GHQ‐12, *M* (*SD*)	12.37 (6.31)	9.42 (4.16)	16.57 (6.84)	11.98 (5.51)	14.58 (6.93)
W9 total loneliness, *M* (*SD*)	4.66 (1.74)	3.27 (0.49)	6.79 (1.28)	6.17 (0.98)	3.74 (0.76)
W10 total loneliness, *M* (*SD*)	4.79 (1.87)	3.40 (0.73)	7.28 (1.25)	4.27 (1.25)	5.75 (1.37)
W11 total loneliness, *M* (*SD*)	4.91 (1.78)	3.64 (0.95)	6.83 (1.54)	4.52 (1.22)	6.15 (1.32)

*Note*: GHQ‐12 has a possible total score of 0–36. Total loneliness scores are based on Three‐Item Loneliness Scale with possible range of 3–9.

### Measures

#### Loneliness

Loneliness was measured at each wave using the Three‐Item Loneliness Scale (Hughes et al., 2004), a brief version of the Revised UCLA Loneliness Scale (Russell et al., 1980). Participants responded to items, ‘How often do you feel you lack companionship’, ‘How often do you feel left out?’ and ‘How often do you feel isolated’, on a 3‐point scale ranging from *hardly ever or never* (1) to *often* (3). This generates a single total loneliness score ranging from 3 to 9, with higher scores indicating greater levels of loneliness. In this study, the scale demonstrated good internal consistency across assessments (Cronbach's *α* = .84, .85 and .84 and McDonald's *ω* = .84, .74 and .84 at Wave 9, 10 and 11, respectively).

#### Psychological distress

Psychological distress was assessed at Wave 9 and Wave 11 using the 12‐item General Health Questionnaire (GHQ‐12; Goldberg & Williams, 1988). Items assess respondents' general functioning and the emergence of distressing feelings, such as: ‘have you been able to concentrate on whatever you're doing?’ and ‘have you recently felt capable of making decisions about things?’. Items have four possible responses: *better than usual* (0), *same as usual* (1), *less than usual* (2) and *much less than usual* (3).

We used two versions of GHQ‐12 scoring. First, we used the standard Likert scoring method (0‐1‐2‐3), yielding a possible total score of 0–36. Higher scores indicated greater levels of psychological distress. This Likert scoring method is consistent with literature examining severity of general psychological distress in late adolescence (Baksheev et al., 2011). The GHQ‐12 showed excellent internal consistency in this study (Cronbach's *α* = .90, McDonald's *ω* = .91). Second, for additional analysis, we used the binary ‘GHQ scoring’ method (0‐0‐1‐1) for Wave 11 psychological distress, where *better than usual* and *same as usual* are scored 0, whereas *less than usual* and *much less than usual* are scored 1. Possible total scores ranged from 0 to 12. A total score of >3 indicates GHQ ‘caseness’, identifying individuals experiencing at least moderate psychological distress (Goldberg et al., 1997).

#### Confounding variables and sensitivity checks

Confounding variables were included in the first step of the regression analyses: sex (male and female), ethnicity (White UK, White other, Black and other) and socio‐economic status indicator of parents' highest educational qualification (no qualification, other qualification, GCSE or equivalent, A‐level or equivalent and higher degree). These variables have been shown to be important to consider in the context of youth loneliness (Maes et al., 2019; Von Soest et al., 2020) and to be associated with psychological distress (Bratter & Eschbach, 2005; Lam et al., 2019; Salk et al., 2017).

Additionally, a sensitivity analysis examined if associations remained robust when adolescents reporting a clinical depression diagnosis were excluded from analysis. This was defined as participants who responded ‘yes’ when asked at Wave 10 if a doctor or other health professional had ever told them that they had clinical depression. Depression diagnosis was an important covariate to consider in analysis of loneliness given the significant correlation between loneliness and self‐report measures of depressive symptoms in adolescents (Dunn & Sicouri, 2022).

### Data analysis

Our analysis was pre‐registered on Open Science Framework (https://osf.io/trvb3). First, we estimated loneliness trajectories using three waves of the Three‐Item Loneliness Scale total score. Several methods of determining clusters in longitudinal data exist (Genolini et al., 2015; Jung & Wickrama, 2008). In this study, we identified and extracted clusters of participants demonstrating distinct loneliness trajectories in R (version 4.3.3) using the R package ‘kml’ (Genolini et al., 2015), based on the k‐means algorithm adapted for longitudinal trajectories. While other clustering methods (e.g., latent class growth analysis) make assumptions about the distribution of the data, the k‐means algorithm is a non‐parametric, partitioning‐based method that makes fewer assumptions about the data and is often used in exploratory analysis, to reduce data complexity and make data more manageable (Morissette & Chartier, 2013). Kml has been applied to identify trajectories of loneliness in adolescents and younger adults (Hutten et al., 2021) and was considered suitable for this study due to its ability to effectively identify distinct subgroups within longitudinal data, as well as its computationally efficient approach and interpretable output (Morissette & Chartier, 2013). Furthermore, kml yields comparable results to latent class growth analysis, identifying similar trajectories of loneliness in youth (Verboon et al., 2022). Missing loneliness data of youth (25.6%) with one missing value for either Wave 10 or Wave 11 total loneliness score were imputed by a two‐step procedure in kml (Genolini et al., 2015). First, linear interpolation was applied where the missing values were replaced with the average of non‐missing, adjacent values. Next, a value was added to this average to make the imputed value follow the shape of the average trajectory.

Consistent with existing literature (Hutten et al., 2021; Qualter et al., 2013; Vanhalst, Goossens, et al., 2013; Vanhalst, Luyckx, et al., 2013), we examined two‐ to six‐cluster solutions. To identify the optimal cluster solution, we considered the quality criteria computed by kml (Genolini et al., 2015), as well as the substantive meaning and the plausibility of the loneliness trajectory clusters based on the developmental periods of adolescence and emerging adulthood, and the longitudinal patterns of loneliness reported in similar research (Ladd & Ettekal, 2013; Qualter et al., 2013; Vanhalst, Goossens, et al., 2013; Vanhalst, Luyckx, et al., 2013). The clusters were then used as an independent variable in subsequent regression analyses.

We examined assumptions of linearity, homoscedasticity and normality using histograms and scatterplots and assessed multicollinearity using correlations and VIF scores. Preliminary analyses included Pearson's correlations to examine associations between the study's key variables. One‐way ANOVA examined unadjusted associations between the loneliness clusters and Wave 11 psychological distress. Significant differences in psychological distress between clusters were identified using Tukey's HSD test for multiple comparisons with an alpha level of *p* < .05.

A hierarchical multiple regression was conducted to examine the association of loneliness cluster with psychological distress while adjusting for confounding variables. Confounding variables were entered in the first step, with loneliness clusters in the second step. Psychological distress at Wave 11 was the dependent variable. Preliminary and regression analyses were completed in IBM SPSS 29.0.

In addition to our pre‐registered analysis, we completed several sensitivity checks to determine the robustness of the associations. We examined if the associations remained significant when participants reporting a diagnosis of clinical depression were excluded from analysis. Next, we examined if the regression results remained significant when adjusting for initial (Wave 9) psychological distress in the first step of the model. Finally, additional exploratory analysis included a binary logistic regression to examine the predictive value of loneliness cluster classification for GHQ caseness.

## RESULTS

### Cluster analysis

**FIGURE 1:**
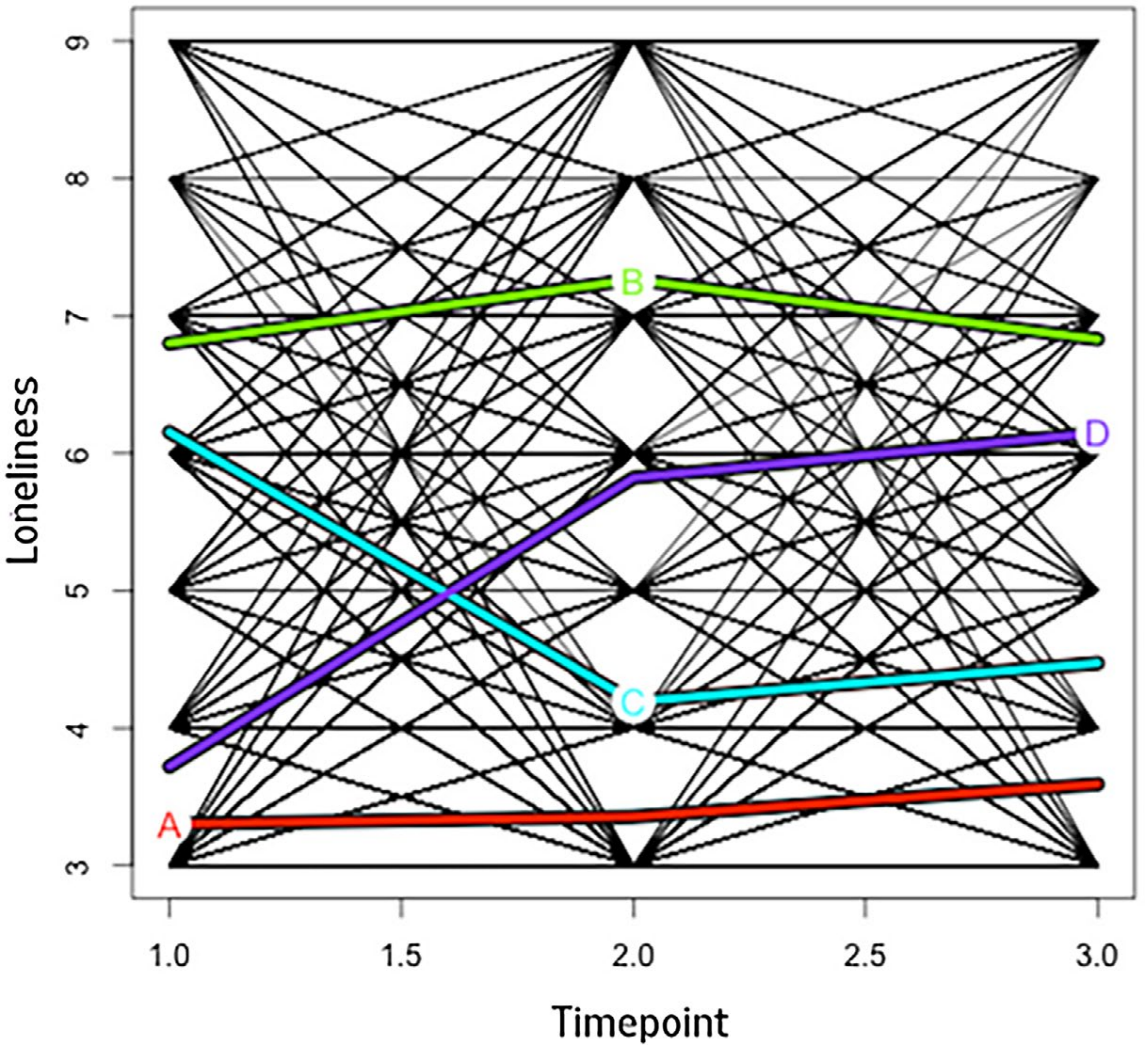
Four‐cluster solution from Kml based on Three‐Item Loneliness Scale total scores. The first cluster (labelled A) can be described as stable low. The second cluster (labelled B) can be described as stable high. The third cluster (labelled C) can be described as moderate decreasing. The fourth cluster (labelled D) can be described as low increasing.

The kml method of longitudinal clustering computed five quality criteria for the two‐ to six‐ cluster solutions, scaled to be between 0 and 1. The criteria did not converge on the same solution. Examination of the model‐fit criteria (BIC and AIC) for the two‐, three‐ and four‐ cluster solutions suggested the solutions were relatively comparable (see Appendix S1). The four‐cluster solution, which had medium values for the quality indices computed by kml, was ultimately chosen as the most optimal solution (Figure 1). This cluster solution aligns with prior research reporting heterogeneity in the developmental trajectories of loneliness in adolescence, identifying four or more distinct clusters (Ladd & Ettekal, 2013; Qualter et al., 2013; Vanhalst, Goossens, et al., 2013; Vanhalst, Luyckx, et al., 2013). These studies highlight that longitudinal patterns of loneliness are likely to show greater variance than simply categorising participants as having stable high or stable low levels (i.e., two‐cluster solution) or high, medium and low levels (i.e., three‐cluster solution) of loneliness. As such, we considered that the four‐cluster solution balanced model parsimony and captured meaningful heterogeneity of loneliness trajectories, potentially identifying more nuanced patterns, including changing loneliness levels.

The first and largest loneliness cluster, labelled stable low (*n* = 337, 40.7%), is characterised by consistently low levels of loneliness across Waves 9, 10 and 11. The second cluster, stable high (*n* = 170, 20.6%), is characterised by relatively high loneliness levels across the three waves. The third cluster, moderate decreasing (*n* = 162, 19.6%), is characterised by moderate loneliness levels at Wave 9 and relatively low loneliness at Waves 10 and 11. The final cluster, low increasing (*n* = 158, 19.1%), is characterised by low levels of loneliness at Wave 9 and moderate loneliness at Waves 10 and 11. Chi‐square tests suggested a significant association between loneliness cluster and sex, *χ* ^2^(3) = 10.697, *p* = .013, Cramer's *V* = .114, and loneliness cluster and ethnicity, *χ* ^2^(9) = 28.97, *p* < .001, Cramer's *V* = .108; in the stable low loneliness cluster, there were more males and youth of other ethnicities, whereas in the stable high loneliness cluster, there were more youth of White than other ethnicity. Parent's highest educational qualification was not significantly associated with loneliness cluster (*p* = .417). See Table 1 for details of demographic characteristics and key variable scores split by loneliness clusters.

### Preliminary analyses

Pearson's correlations (see Table 2) showed a significant association between total loneliness score at each wave with Wave 11 psychological distress (*r* = .33, .40 and .56, for Waves 9, 10 and 11, respectively). In unadjusted analyses, psychological distress at Wave 11 significantly differed between loneliness clusters, *F*(3, 663) = 56.450, *p* < .001, *η* ^2^ = .203. Post‐hoc Tukey's test found that participants in the stable high (*p* < .001, *M* = 16.57, *SD* = 6.84), moderate‐decreasing (*p* < .001, *M* = 11.98, *SD* = 5.51) and low‐increasing (*p* < .001, *M* = 14.58, *SD* = 6.93) loneliness clusters had on average, significantly higher psychological distress than those in the stable low cluster (*M* = 9.42, *SD* = 4.16).

**TABLE 2:** Correlations between main study variables.

Variable	1	2	3	4	5	6
1. W9 total loneliness	1					
2. W10 total loneliness	.47**	1				
3. W11 total loneliness	.39**	.56**	1			
4. W9 total GHQ score	.51**	.36**	.27**	1		
5. W11 total GHQ score	.33**	.40*	.56**	.41**	1	
6. Sex	.09*	.07*	.05	.10**	.16**	1

** Significant at .01 level.

* Significant at .05 level.

### Regression analysis

To examine the hypothesis that loneliness trajectories characterised by consistently high levels of loneliness will report greater psychological distress, we conducted a hierarchical multiple regression presented in Table 3. The confounding variables of sex, ethnicity and parents' highest educational qualification were entered in the first step of the model. Loneliness trajectory clusters were included in the second step. The overall model significantly predicted psychological distress, *F*(11, 623) = 16.831 *p* < .001, adjusted *R* ^2^ = .215. The addition of the loneliness trajectory clusters in the second step explained 18.9% of the variance in psychological distress (adjusted Δ*R* ^2^ = .189). Compared to stable low loneliness, stable high (*β* = .449, *p* < .001), moderate‐decreasing (*β* = .146, *p* < .001) and low‐increasing (*β* = .317, *p* < .001) loneliness clusters were significantly associated with a higher risk of psychological distress following full adjustment.

**TABLE 3:** Regression analysis predicting psychological distress.

Variable	*B*	*β*	*t*	*p*	95% CI	
					LL	UL
Sex (male as reference)	1.825	.144	4.033	<.001	.936	2.713
Ethnicity (White UK as reference)						
White other	.572	.020	.555	.579	−1.450	2.594
Black	−1.248	−.041	−1.143	.253	−3.393	.896
Other	.046	.003	.084	.933	−1.031	1.123
Parent's highest educational qualification (no qualification as reference)						
Other qualification	.119	.004	.082	.935	−2.736	2.974
GCSE or equivalent	.248	.015	.198	.843	−2.214	2.710
A‐level or equivalent	−.737	−.046	−.586	.558	−3.208	1.734
Higher degree	.177	.014	.149	.881	−2.152	2.506
Loneliness cluster (stable low loneliness as reference)						
Stable high	7.000	.449	11.474	<.001	5.802	8.198
Moderate decreasing	2.323	.146	3.770	<.001	1.113	3.532
Low increasing	5.077	.317	8.173	<.001	3.857	6.297

### Additional analyses

#### Sensitivity analyses

First, to determine if having a diagnosis of clinical depression impacted the findings, the analysis was conducted with these participants excluded (*n* = 27). Estimates were virtually unchanged. Next, a sensitivity analysis adjusting for Wave 9 psychological distress indicated that the association between the moderate‐decreasing loneliness trajectory and psychological distress was non‐significant following adjustment. The overall interpretation of associations between stable high‐ and low‐increasing loneliness trajectories and psychological distress remained the same. See Appendix S2 for sensitivity analysis tables.

#### Logistic regression predicting GHQ caseness

A total of 189 (22.9%) participants met the cut‐off score for GHQ caseness. A binary logistic regression was conducted to examine whether loneliness cluster predicted GHQ caseness while statistically controlling for confounding variables. Overall, the model was statistically significant (*χ* ^2^[11] = 129.750, *p* < .001), accounting for 26.6% (Nagelkerke *R* ^2^) of the variance in GHQ caseness and correctly classifying 75.3% of participants. Compared to those with a stable low loneliness trajectory, participants with stable high (OR = 11.290, 95% CI [6.44, 19.79]), moderate‐decreasing (OR = 2.677, 95% CI [1.48, 4.86]) and low‐increasing (OR = 7.28, 95% CI [4.18, 12.67]) loneliness trajectories were significantly more likely to have psychological distress caseness. See Appendix S3 for full details.

## DISCUSSION

Using a population‐based dataset, this study identified clusters of loneliness in youth demonstrating distinct longitudinal trajectories and examined their association with psychological distress. As hypothesised, youth displaying persistent, or recurrent, high levels of loneliness were at greatest risk of psychological distress, compared to stable low loneliness. Findings also showed a significant association between low‐increasing loneliness and psychological distress. Following adjustment for initial levels of psychological distress in a sensitivity analysis, the moderate‐decreasing loneliness trajectory was not significantly associated with psychological distress. This study extends the limited existing research by providing evidence for an association between longitudinal trajectories of loneliness and poorer mental health outcomes in a sample of UK late adolescents.

The results of this study suggest heterogeneity in the development of loneliness in youth. We identified four trajectory clusters across three annual waves representing youth who demonstrated (1) very low or no loneliness (i.e., stable low), (2) consistent high loneliness (i.e., stable high), (3) a decreasing loneliness trend (i.e., moderate decreasing) and (4) an increase in loneliness (i.e., low increasing). Consistent with previous loneliness trajectory research in late adolescents and emerging adults (Hutten et al., 2021; Lin & Chiao, 2022; Qualter et al., 2013; Vanhalst, Goossens, et al., 2013; Vanhalst, Luyckx, et al., 2013), the largest cluster of youth in this study reported a stable low loneliness trajectory. This suggests that a proportion of youth manage to navigate the increased autonomy and developmental changes in social relationships that characterise this life stage quite well (Arnett, 2024; Laursen & Hartl, 2013), at least in terms of loneliness.

However, some youth demonstrated stable, relatively high levels of loneliness across assessments. This stable high trajectory represented the experience of loneliness for more youth in our study (20.6%) than previous research reporting a stable high loneliness pattern in 3% of participants from the ages of 15–20 years (Vanhalst, Goossens, et al., 2013; Vanhalst, Luyckx, et al., 2013) and 13.7% of adolescents from the ages of 12 to 18 years (Ladd & Ettekal, 2013). This variability may be due to the extended age ranges included in previous studies or because of differences in loneliness measurement and the levels of loneliness considered to be high. Although participants in our stable high cluster demonstrated consistently higher loneliness levels compared to other trajectories, they did not report very high levels on the loneliness measure. Nonetheless, this trajectory was associated with psychological distress demonstrating a medium effect size. Youth experiencing persistent loneliness may be a risk group, not solely due to elevated loneliness levels but also due to their risk of poorer mental health outcomes. The low‐increasing trajectory was another group at increased risk of psychological distress, compared to the stable low cluster. Our results suggest almost one‐fifth (19.1%) of youth reported relatively low loneliness levels at baseline, similar to those with stable low loneliness, but reported increased loneliness over follow‐up waves. A twin study of adolescents reported that while genetic influences largely explained the stability of loneliness levels over time, non‐shared environmental factors played a substantial role in explaining increases in loneliness (Matthews et al., 2016). Individual differences in loneliness may arise due to disruptions to social networks linked to transitions that occur for some adolescents, but not all. For example, moving to a new city for employment or university.

On the other hand, some youth can overcome loneliness, as suggested by our finding of a moderate‐decreasing trajectory, reflecting transient loneliness. Transient loneliness is likely to be common in late adolescence and emerging adulthood due to the challenges of social reorientation during this life stage; developmental shifts in social companions include prioritising peer relationships over family and spending more time with romantic partners in later adolescence and emerging adulthood (Laursen & Hartl, 2013). Qualitative research suggests that youth themselves view transient loneliness as a consequence of normative development and typical social transitions during this period (Kirwan et al., 2023). Taking a conservative approach to increase the likelihood that associations are a result of following the loneliness trajectory and not earlier levels of the outcome, we adjusted for initial levels of psychological distress in additional analyses. Our findings indicate that, compared to stable low loneliness, the moderate‐decreasing trajectory was not significantly associated with psychological distress following adjustment. This finding is at odds with other research that has indicated that youth demonstrating a decreasing loneliness trend may still be at increased risk of depression, compared to consistently low loneliness (Hutten et al., 2021; Schinka et al., 2013). Additionally, recurring experiences of loneliness during adolescence may not necessarily translate to a cumulative risk of poorer mental health, but experiencing loneliness during early adolescence may have long‐term implications, regardless of whether it recurs (Matthews et al., 2023). Future longitudinal research should extend this work in late adolescence and emerging adulthood and examine the relevance of the scar hypothesis (Rohde et al., 1990) for loneliness, where initially higher levels that subsequently decrease are still related to poorer outcomes. It is possible that the youth demonstrating a moderate‐decreasing trajectory in our study did not experience very intense loneliness, despite its relatively high frequency at Wave 9. Therefore, future research should examine not only the duration but also the intensity of loneliness during late adolescence and emerging adulthood to determine its association with psychological distress.

Why some youth demonstrate chronic loneliness, while others manage to resolve temporary feelings, is likely to be complex. Chronic loneliness over the life course may be rooted in early life experiences, such as poor parental bonding (Burns et al., 2022). The evolutionary theory of loneliness (Cacioppo et al., 2006) is one approach suggesting potential mechanisms for the development of chronic loneliness in youth. This model suggests that although distressing, loneliness is adaptive in the short term to signal low‐quality, threatened or absent social connections. As such, transient loneliness serves as motivation to mend deficient connections, activating the reaffiliation motive whereby lonely individuals become hypervigilant for social cues (Qualter et al., 2015). Some youth recognise and capitalise on opportunities for social connection, effectively alleviating loneliness. However, loneliness can cause individuals to interpret social cues as threatening due to the perceived lack of belonging and protection from others. Lonely youth may withdraw from social interaction to avoid rejection, leading to missed opportunities for connection and positive interactions, thereby perpetuating chronic loneliness. This process may be particularly relevant during adolescence and emerging adulthood (Goossens, 2018). The uncertainty associated with typical social challenges and transitions may heighten sensitivity to ambiguous social cues, and the increased self‐focus characteristic of emerging adulthood (Arnett, 2024) may impact efforts to reconnect with others, fostering a cycle of withdrawal and loneliness. Given the distress associated in youth and potential for chronic loneliness to shape poor outcomes throughout the lifespan, including premature mortality (Holt‐Lunstad et al., 2015), future research should focus efforts on identifying youth most at risk for developing persistent loneliness.

### Limitations

A strength of this study includes the use of population‐based longitudinal data, which has been identified as lacking in loneliness research in youth (Kirwan et al., 2024). However, some limitations and considerations are necessary when interpreting our results. First, this study was conducted in the United Kingdom only, limiting the generalisability of the results to youth in other geographical regions. Future research should explore loneliness trajectories in population‐based samples of youth from other countries to allow for comparisons with our findings. Second, some Wave 11 data were collected during the COVID‐19 pandemic lockdowns in the United Kingdom, which may have resulted in elevated loneliness or psychological distress. However, other research using UKHLS data suggested minimal increases in loneliness and psychological distress during COVID‐19 (Milicev et al., 2023), and a systematic review reported that mental health symptoms generally returned to pre‐pandemic levels by mid‐2020 (Robinson et al., 2022). Third, although the Three‐Item Loneliness Scale (Hughes et al., 2004) is widely used in large‐scale surveys and showed good reliability in this study, such brief measures may not fully capture the complexity of loneliness (Mund et al., 2023). Fourth, we used k‐means longitudinal clustering because of its usability and computational efficiency to produce an interpretable output of clusters (Morissette & Chartier, 2013). However, there is no ‘gold standard’ method for clustering in longitudinal data, and other methods like latent class growth analysis may be suitable alternatives. Although previous research has suggested that kml and a latent growth method yield similar results (Verboon et al., 2022), we acknowledge that different methods may produce clusters that vary due to differences in model assumptions and computational approaches. Additionally, our four‐cluster trajectory solution reflects the variation in loneliness that is well established in previous research with similar age groups (Qualter et al., 2013; Vanhalst, Goossens, et al., 2013; Vanhalst, Luyckx, et al., 2013). However, loneliness trajectories in this sample were not clearly distilled into an optimal cluster solution according to model fit indices; a two‐ or three‐ cluster solution was also an acceptable fit for the data. While a consistent loneliness measure across three annual time points was a strength of this study, the loneliness clusters would be more robust if the number of measurement waves were larger.

In this study, we cannot conclude the directionality of the relationship between loneliness and psychological distress, which may be bidirectional (Vanhalst et al., 2012). Loneliness may lead to psychological distress through social withdrawal and increased rumination (van Winkel et al., 2017; Vanhalst, Goossens, et al., 2013; Vanhalst, Luyckx, et al., 2013). Conversely, psychological distress might contribute to loneliness by reducing social interaction and fostering negative cognitive biases that lead to the misinterpretation of social cues (Bangee et al., 2014; Beevers et al., 2019; Elmer & Stadtfeld, 2020). Furthermore, these mechanisms could also create a self‐reinforcing cycle, where loneliness and psychological distress exacerbate each other over time (Vanhalst et al., 2012). In research among older adults, loneliness predicted subsequent depressive symptoms but not vice versa (Cacioppo et al., 2010). While these results support our approach, future research should examine the potential bidirectional relations between trajectories of loneliness and psychological distress specifically in youth. Although a sensitivity analysis was conducted to assess whether the associations remained significant after excluding participants with a clinical diagnosis of depression, few such cases were in this sample. This may be due to unreliable self‐reporting or because some youth experiencing symptoms have not yet sought a clinical diagnosis. Nonetheless, future studies should control for clinical depression diagnoses to better understand the independent associations between loneliness trajectories and psychological distress.

## CONCLUSION

This study examined the presence of clusters of youth demonstrating distinct longitudinal trajectories of loneliness and examined the association of loneliness trajectories with psychological distress. Our findings indicate that stable, relatively high loneliness levels during late adolescence and emerging adulthood may be detrimental to mental health. Chronically lonely adolescents who require support may benefit from interventions aimed at addressing loneliness and psychological distress. Results also suggest that individuals demonstrating trajectories characterised by increasing loneliness levels may also be at risk of poorer mental health. Therefore, a focus on supporting social connection during late adolescence may be beneficial for youth mental health.

## AUTHOR CONTRIBUTIONS

**Emma M. Kirwan:** Conceptualisation; funding acquisition; writing – original draft; methodology; writing – review and editing; formal analysis; data curation. **Martina Luchetti:** Conceptualisation; writing – original draft; writing – review and editing; formal analysis; methodology. **Annette Burns:** Conceptualisation; writing – original draft; writing – review and editing; methodology. **Páraic S. O'Súilleabháin:** Conceptualisation; writing – original draft; writing – review and editing; methodology. **Ann‐Marie Creaven:** Conceptualation; methodology; writing – original draft; writing – review and editing; supervision.

## CONFLICT OF INTEREST STATEMENT

The authors declare no conflicts of interest with respect to the research, authorship and/or publication of this article.

## ETHICAL APPROVAL

Ethical approval for UKHLS was granted by the Essex Ethics Committee (Understanding Society, n.d.).

## Supporting information


Appendix S1.–S3.

## Data Availability

The data used in the current study are publicly available from the UK Data Service: https://beta.ukdataservice.ac.uk/datacatalogue/studies/study?id=6614.
