# Supplementary material for: Loneliness trajectories and psychological distress in youth: Longitudinal evidence from a population‐based sample
Source: Br J Dev Psychol. 2024 Nov 16;43(1):190–204. doi: 10.1111/bjdp.12533 (PMC11823292; doi:10.1111/bjdp.12533)
Supplement: Supplementary file 1 — Appendix S1.–S3. [file BJDP-43-190-s001.docx]

**Supplementary Materials**

**Loneliness trajectories and psychological distress in youth: Longitudinal evidence from a population-based sample**

**Appendix S1**

**Model Fit Criteria (BIC And AIC) For the Two-, Three-, and Four- Cluster Solutions**

**Table S1.**

*BIC and AIC Values for the Two-, Three-, and Four-Cluster Trajectory Solutions*

| Cluster solution | BIC | AIC |
| --- | --- | --- |
| Two-cluster solution | -8624.679 | -8591.654 |
| Three-cluster solution | -8610.818 | -8563.910 |
| Four-cluster solution | -8581.215 | -8519.883 |

**Appendix S2**

**Sensitivity Analyses Tables**

**Table S2.1.**

*Regression Analysis Predicting Wave 11 Psychological Distress Adjusted for Wave 9 Psychological Distress*

| Variable |  |  |  |  | 95% CI | |
| --- | --- | --- | --- | --- | --- | --- |
|  | B | *β* | t | *p* | LL | UL |
| Wave 9 Psychological Distress | .279 | .293 | 7.518 | <.001 | .206 | .352 |
| Sex (male as reference) | 1.348 | .106 | 3.077 | .002 | .488 | 2.209 |
| Ethnicity (White UK as reference) | | | | | | |
| Other White | .817 | .028 | .828 | .408 | -1.121 | 2.756 |
| Black | -.828 | -.027 | -.790 | .430 | -2.886 | 1.230 |
| Other | .218 | .015 | .414 | .679 | -.815 | 1.251 |
| Parent’s highest educational qualification (no qualification as reference) | | | | | | |
| Other qualification | -.122 | -.005 | -.088 | .930 | -2.859 | 2.614 |
| GCSE or equivalent | .227 | .014 | .189 | .850 | -2.132 | 2.586 |
| A-level or equivalent | -.651 | -.041 | -.540 | .589 | -3.019 | 1.717 |
| Degree | .108 | .009 | .095 | .925 | -2.124 | 2.340 |
| Loneliness cluster (stable low loneliness as reference) | | | | | | |
| Stable high | 4.878 | .313 | 7.514 | <.001 | 3.603 | 6.153 |
| Moderate decreasing | .838 | .053 | 1.346 | .179 | -.384 | 2.060 |
| Low increasing | 4.665 | .291 | 7.803 | <.001 | 3.491 | 5.839 |

**Results of Hierarchical Regression Analysis predicting psychological distress from loneliness trajectories with participants with self-reported clinical depression diagnosis (*n* = 27) excluded from analysis**

A hierarchical regression analysis was conducted with confounding variables sex, ethnicity, and parents’ highest educational qualification in step 1. Loneliness trajectory clusters were entered in step 2.

The overall model significantly predicted psychological distress, *F*(11, 558) = 13.816, *p* < .001, adjusted R^2^ = .199). Compared to stable low loneliness, stable high (*β* = .423, *p* < .001), moderate decreasing (*β* = .136, *p* = .001), and low increasing (*β* = .302, *p* < .001) loneliness clusters were significantly associated with a higher risk of psychological distress following adjustment.

**Table S2.2.**

| Variable |  |  |  |  | 95% CI | |
| --- | --- | --- | --- | --- | --- | --- |
|  | B | *β* | t | *p* | LL | UL |
| Sex (male as reference) | 1.575 | .126 | 3.310 | <.001 | .640 | 2.509 |
| Ethnicity (White UK as reference) | | |  | | | |
| White other | .906 | .032 | .826 | .409 | -1.248 | 3.060 |
| Black | -2.314 | -.071 | -1.857 | .064 | -4.761 | .133 |
| Other | -.118 | -.008 | -.204 | .838 | -1.248 | 1.013 |
| Parent’s highest educational qualification (no qualification as reference) | | | | | | |
| Other qualification | -1.269 | -.047 | -.833 | .405 | -4.260 | 1.722 |
| GCSE etc. | .294 | .018 | .227 | .820 | -2.246 | 2.833 |
| A-level etc. | -.652 | -.042 | -.506 | .613 | -3.187 | 1.882 |
| Higher degree | .246 | .020 | .202 | .840 | -2.143 | 2.634 |
| Loneliness cluster (stable low loneliness as reference) | | | | | | |
| Stable high | 6.659 | .423 | 10.185 | <.001 | 5.375 | 7.943 |
| Moderate decreasing | 2.135 | .136 | 3.305 | .001 | .866 | 3.404 |
| Low increasing | 4.773 | .302 | 7.352 | <.001 | 3.497 | 6.048 |

*Regression Analysis Predicting Wave 11 Psychological Distress with Participants Reporting Clinical Depression Diagnosis (n = 27) Excluded from Analysis*

**Appendix S3**

**Results of binary logistic regression predicting GHQ caseness from loneliness trajectory**

A total of 189 (22.9%) participants met the cut-off score for GHQ caseness. A binary logistic regression was conducted to examine if loneliness cluster predicted GHQ caseness while statistically controlling for confounding variables.

The Hosmer and Lemeshow test suggested that the data was an acceptable fit for the model (*χ*^2^[7] = 3.917, *p* = .789). Overall, the model was statistically significant (*χ*^2^[11] = 129.750, p < .001), accounting for 26.6% (Nagelkerke R^2^) of the variance in GHQ caseness and correctly classifying 75.3% of participants. Compared to those with a stable low loneliness trajectory, participants with stable high loneliness were over 6 times more likely to meet the cut-off for significant psychological distress. Moderate decreasing and low increasing loneliness trajectories were 1.5 and 4 times more likely to have psychological distress caseness, respectively. See Table S3.

**Table S3.**

*Binary Logistic Regression Predicting GHQ Caseness from Loneliness Cluster*

| Variable |  |  | 95% CI | |  |
| --- | --- | --- | --- | --- | --- |
|  | B (SE) | OR | LL | UL | *p* |
| Sex (male as reference) | .914 (.208) | 2.493 | 1.659 | 3.747 | < .001 |
| Ethnicity (White UK as reference) | |  |  |  |  |
| White other | .079 (.455) | 1.082 | .444 | 2.638 | .862 |
| Black | -.198 (.512) | .821 | .301 | 2.240 | .700 |
| Other | .231 (.254) | 1.259 | .765 | 2.073 | .365 |
| Parent’s highest educational qualification (no qualification as reference) | | | |  |  |
| Other qualification | 1.414 (.888) | 4.111 | .722 | 23.416 | .111 |
| GCSE etc. | 1.481 (.818) | 4.397 | .885 | 21.835 | .070 |
| A-level etc. | .924 (.828) | 2.519 | .497 | 12.751 | .264 |
| Higher degree | 1.362 (801) | 3.902 | .811 | 18.768 | .089 |
| Loneliness cluster (stable low loneliness as reference) | | | |  |  |
| Stable high | 2.424 (.286) | 11.290 | 6.443 | 19.785 | <.001 |
| Moderate decreasing | .985 (.304) | 2.677 | 1.476 | 4.858 | .001 |
| Low increasing | 1.985 (.283) | 7.280 | 4.184 | 12.669 | <.001 |
